# Analysis of health service utilization and influencing factors due to COVID-19 in Beijing: a large cross-sectional survey

**DOI:** 10.1186/s12961-024-01118-6

**Published:** 2024-03-04

**Authors:** Jiawei Zhang, Zhihu Xu, Xia Wei, Yaqun Fu, Zheng Zhu, Quan Wang, Qingbo Wang, Qing Liu, Jing Guo, Yuantao Hao, Li Yang

**Affiliations:** 1 Department of Health Policy and Management, Peking University School of Public Health, 38 Xueyuan Road, Haidian District, Beijing, 100191 China; 2 Department of Occupational and Environmental Health Sciences, Peking University School of Public Health, 38 Xueyuan Road, Haidian District, Beijing, 100191 China; 3 Department of Health Services Research and Policy, London School of Hygiene & Tropical Medicine, 15-17 Tavistock Place, London, WC1H 9SH United Kingdom; 4 Brown School, Washington University in St. Louis, St. Louis, Missouri 63130 United States of America; 5 General Practice Department, Second Outpatient Section, Peking University Third Hospital, Xisanqi Street, Haidian District, Beijing, 100096 China; 6 Center for Public Health and Epidemic Preparedness and Response, Peking University, 38 Xueyuan Road, Haidian District, Beijing, 100191 China

**Keywords:** Health service utilization, COVID-19, Primary healthcare services, Internet-based healthcare

## Abstract

**Background:**

In the wake of China’s relaxed zero-COVID policy, there was a surge in coronavirus disease 2019 (COVID-19) infections. This study aimed to examine the infection status and health service utilization among Beijing residents during a widespread outbreak, and to explore the factors that affected utilization of health services due to COVID-19.

**Methods:**

A cross-sectional survey was conducted among Beijing residents from 13 January to 13 February 2023, collecting information on socio-demographic characteristics, health behaviours, COVID-19 infection status, utilization of health services and depressive symptoms. Multivariate Tobit regression was used for data analysis.

**Results:**

Among the 53 924 participants, 14.7% were older than 60 years, 63.7% were female and 84.8% were married. In total, 44 992 of the 53 924 individuals surveyed (83.4%) contracted COVID-19 during 2020–2023, and 25.2% (13 587) sought corresponding health services. The majority of individuals (85.6%) chose in-person healthcare, while 14.4% chose internet-based healthcare. Among those who chose in-person healthcare, 58.6% preferred primary healthcare institutions and 41.5% were very satisfied with the treatment. Factors affecting health service utilization include being female (*β* = −0.15, *P* < 0.001), older than 60 years (*β* = 0.23, *P* < 0.01), non-healthcare workers (*β* = −0.60, *P* < 0.001), rich self-rated income level (*β* = 0.59, *P* < 0.001), having underlying disease (*β* = 0.51, *P* < 0.001), living alone (*β* = −0.19, *P* < 0.05), depressive symptoms (*β* = 0.06, *P* < 0.001) and healthy lifestyle habits, as well as longer infection duration, higher infection numbers and severe symptoms.

**Conclusion:**

As COVID-19 is becoming more frequent and less severe, providing safe and accessible healthcare remains critical. Vulnerable groups such as the elderly and those with underlying conditions need reliable health service. Prioritizing primary healthcare resources and online medical services have played a vital role in enhancing resource utilization efficiency.

**Supplementary Information:**

The online version contains supplementary material available at 10.1186/s12961-024-01118-6.

## Introduction

In December 2019, a novel coronavirus emerged, causing the coronavirus disease 2019 (COVID-19) pandemic [1]. After its emergence, COVID-19 rapidly escalated into a global public health crisis. Given the virus’s mutation and increasing vaccination coverage, prevention and control strategies implemented by various regions have begun to adjust. On 7 December 2022, the Chinese government introduced 10 optimized measures for epidemic prevention and control that involve scientifically precise delineation of high-risk areas and prohibited mass testing by administrative regions [2]. Simultaneously with the adjustments made, the number of COVID-19 infections started to grow rapidly [3, 4], accompanied by a concomitant increase in demand for medical care in China [5]. An online survey of the Macau population found that more than 70% of residents were infected within 3 weeks after the change in the zero-COVID policy [4].

Some researchers used epidemic nowcast framework to estimate that the peak of infection in Beijing was reached on 22 December, when about 76% of the residents had been infected. By 31 January 2023, 92.3% might have been already infected [6]. This has placed a huge burden on the healthcare system, with short-term drug shortages and medical staff working even when infected [7].

The utilization of healthcare services by patients has been severely impacted since the emergence of COVID-19, with a falling trend recorded in both COVID-19- and non-COVID-19-related patient visits. After the COVID-19 outbreak, medical facilities across China have experienced substantial reductions in both health facility visits and inpatient volume. As of June 2020, a majority of the metrics used to gauge the healthcare sector’s performance have not yet rebounded to their pre-pandemic levels [8]. Coinciding with these trends, a study has documented significant cuts in total healthcare expenditure and utilization in China [9]. In the Netherlands, oncology care and cancer screening programs have been neglected as healthcare services respond adequately to the surge in patients with COVID-19 [10]. At the beginning of the COVID-19 pandemic, the WHO determined that the pandemic was affecting not only physical, but also mental health [11, 12], but the utilization of psychological services has significantly decreased [13, 14]. The utilization of health services for non-communicable diseases has also been notably affected [8]. Most of the studies on service utilization during the COVID-19 pandemic have focussed on the early stages of the outbreak when prevention and control strategies were more stringent. However, there have been few studies on health service utilization after relaxation of the dynamic zero-COVID policy.

After the adjustment of prevention and control policies, no research has been conducted to analyse health service utilization of residents in China. Therefore, to fill this research gap regarding the utilization of health services during a large-scale infection period, this study conducted a comprehensive questionnaire survey in Beijing requesting that residents analyse health service utilization due to COVID-19 and their influencing factors to explore the characteristics and differences among different age groups, education levels, economic conditions and so on. This study provided valuable evidence for ensuring the provision of health services to residents after relaxation of the dynamic zero-COVID policy.

## Methods

### Study design

Our study employed electronic questionnaires (the famous online questionnaire platform Wenjuanxing) to investigate personal characteristics, infection, symptom manifestations and health service utilization among community residents in Beijing following the relaxation of the dynamic zero-COVID policy from 13 January to 13 February 2023, after the major wave of COVID-19 infections. Cluster random sampling was utilized to ensure sample representativeness. Specifically, 16 administrative districts in Beijing were identified as the primary sampling frame, and between 8 and 42 primary healthcare institutions were randomly selected from each district in proportion to population size, resulting in a total of 293 primary healthcare institutions being included in the study. Subsequently, 3–5 family doctors were selected from each primary healthcare institution, and each doctor surveyed 40–50 contracted residents, resulting in a total of 60 039 participants. Following exclusion of invalid responses and missing data, a total of 53 924 questionnaires were deemed suitable for analysis.

## Measurements

### Health service utilization

One section of the questionnaire pertained to the utilization of health services due to COVID-19, including the number of visits made for COVID-19 symptoms, the number of days with COVID-19 symptoms before seeking health services (day 1, day 2, day 3, days 4–5, more than 5 days or unclear), the form of health consultation (online or in-person), the preferred type of medical institution (primary healthcare institution, first-level hospital, secondary hospital or tertiary hospital) and the waiting time for medical treatment (within 15 min, 15–30 min, 30–60 min, 1–2 h, 2–4 h, 4–6 h or more than 6 h).

There were also questions regarding the completion of the medical consultation, including whether hospitalization was required due to the severity of COVID-19 symptoms, the improvement in symptoms after treatment (cured, significantly improved, slightly improved, not improved or worsened) and satisfaction with treatment services (not satisfied, not very satisfied, basically satisfied, satisfied or very satisfied).

### COVID-19 infection and symptoms

Participants were also queried regarding their COVID-19 infection status. Specifically, we requested data on whether they had been infected, as well as the number of times they had been infected (none, once, twice, three times or more). For those who indicated having experienced an infection, we asked for details on symptom duration (10 days or less, 11–20 days, 21–30 days, more than 30 days) and severity (asymptomatic, mild, moderate, severe) during their most recent bout. The questionnaire also investigated the intention (yes or no) of infected people to seek health services for COVID-19 symptoms.

### Demographic and health-related characteristics

The questionnaire utilized in our study entailed the collection of various socio-demographic parameters from participants. These included gender, age (18–30, 31–45, 46–60, older than 60), relative income status (poor, average or rich), marital status (married or not married, including unmarried, divorced and widowed), education level (junior high school degree and below, high school degree or college and above), occupation type (healthcare workers or non-healthcare workers), medical insurance coverage (urban employee basic medical insurance, urban and rural residents basic medical insurance, free medical care or medical out of pocket) and household size (live alone or not). Additionally, we also gathered data on several health-related factors, such as the presence of underlying diseases (yes or no), sleep quality (very poor, poor, fair, good or very good), smoking (yes or no), alcohol consumption (yes or no) and exercise habits (yes or no). The distance to the nearest medical facility from their home (less than 1 km, 1–3 km, 3 km and further) and the influence of COVID-19 on healthcare seeking (yes or no) was also investigated.

### Psychological scale measurement

The most frequently used screening tool to detect depressive symptoms in primary care is the Patient Health Questionnaire-9 (PHQ-9) [15, 16]. Composed of nine questions and a scoring system that ranges from 0 to 27, it has been validated for measuring the severity of depressive symptoms according to five grades: no depression (scores of 0–4), mild depression (5–9), moderate depression (10–14), moderately severe depression (15–19) and severe depression (20–27) [17, 18]. The prevalence of COVID-19 can potentially result in anxiety and depression among the population. In this study, the Cronbach’s *α* of this scale is 0.96.

### Statistical analysis

Categorical variables involved in the questionnaire were depicted by frequencies and percentages, and continuous variables were shown as means and standard deviations.

The Tobit model, first introduced in 1958, is a widely used regression analysis method in the fields of economics, sociology and medicine, designed to handle dependent variables that are truncated or censored. When the dependent variable is truncated due to certain constraints with its value set between upper and lower limits or when some observations have zero values, the Tobit model can correct the bias estimate from the ordinary least squares (OLS) regression [19]. The dependent variable in the Tobit regression in this study was the number of visits made for COVID-19 symptoms, and the independent variables were personal socio-economic and health-related indicators, symptom severity and psychological scale results. The statistical analyses were performed using R software (version 4.0.3, R Foundation). We used R package “censReg” to conduct Tobit regression [20]. Two-tailed test with *P*-value < 0.05 was considered statistically significant.

## Results

### Participant characteristics

Table 1 summarizes the socio-demographic characteristics of the participants. Amongst the 53 924 participants, 14.7% were older than 60 years, with the highest concentration of people aged from 31 to 45 years (40.3%). Amongst all participants, 63.7% were female, 84.8% were married, 4.8% lived alone, 86.8% were non-healthcare workers and 59.0% had college or higher education. A total of 11 599 (21.5%) participants self-rated their income as poor, 41 112 (76.2%) as average and 1213 (2.2%) as rich. With regard to health behaviours, 19.1% of participants smoked, 41.7% drank alcohol and 29.8% did not exercise regularly. A total of 5.4% of participants reported excellent sleep quality, while 35.4% had underlying diseases. Overall, 36.1% participants reported an impact on health service utilization due to COVID-19. This effect stemmed from concerns about infection and the crowded nature of healthcare facilities, making routine medical appointments challenging. The average score of the participant's PHQ-9 questionnaire was 6.01 (standard deviation was 6.43).

**Table 1:** Socio-demographic characteristics of all, uninfected, infected but not utilizing health services, infected and utilizing health services participants

Characteristic	Total	Uninfected	Infected	
			Not utilizing health services	Utilizing health services
*n*	53 924	8932	31 405	13 587
Age [years, *n*(%)]				
18–30	8213 (15.2)	1091 (12.2)	5149 (16.4)	1973 (14.5)
31–45	21 754 (40.3)	2658 (29.8)	13 950 (44.4)	5146 (37.9)
46–60	16 052 (29.8)	3075 (34.4)	8741 (27.8)	4236 (31.2)
Older than 60	7905 (14.7)	2108 (23.6)	3565 (11.4)	2232 (16.4)
Gender [*n*(%)]				
Female	34 344 (63.7)	5151 (57.7)	20 307 (64.7)	8886 (65.4)
Male	19 580 (36.3)	3781 (42.3)	11 098 (35.3)	4701 (34.6)
Marital status [*n*(%)]				
Married	45 742 (84.8)	7425 (83.1)	26 669 (84.9)	11 648 (85.7)
Not married	8182 (15.2)	1507 (16.9)	4736 (15.1)	1939 (14.3)
Household size [*n*(%)]				
Living alone	2585 (4.8)	689 (7.7)	1356 (4.3)	540 (4.0)
Not living alone	51 339 (95.2)	8243 (92.3)	30 049 (95.7)	13 047 (96.0)
Occupation type [*n*(%)]				
Healthcare workers	7110 (13.2)	723 (8.1)	4115 (13.1)	2272 (16.7)
Non-healthcare workers	46 814 (86.8)	8209 (91.9)	27 290 (86.9)	11 315 (83.3)
Medical insurance [*n*(%)]				
Urban employee basic medical insurance	40 098 (74.4)	5756 (64.4)	23 963 (76.3)	10 379 (76.4)
Urban and rural residents basic medical insurance	8455 (15.7)	2006 (22.5)	4507 (14.4)	1942 (14.3)
Free medical care	2558 (4.7)	525 (5.9)	1398 (4.5)	635 (4.7)
Medical out of pocket	2813 (5.2)	645 (7.2)	1537 (4.9)	631 (4.6)
Education [*n*(%)]				
Junior high school degree and below	10 443 (19.4)	2464 (27.6)	5445 (17.3)	2534 (18.7)
High school degree	11 654 (21.6)	2348 (26.3)	6417 (20.4)	2889 (21.3)
College and above	31 827 (59.0)	4120 (46.1)	19 543 (62.2)	8164 (60.1)
Relative income status [*n*(%)]				
Poor	11 599 (21.5)	1963 (22.0)	6813 (21.7)	2823 (20.8)
Average	41 112 (76.2)	6758 (75.7)	23 948 (76.3)	10 406 (76.6)
Rich	1213 (2.2)	211 (2.4)	644 (2.1)	358 (2.6)
Smoking [*n*(%)]				
Yes	10 292 (19.1)	2231 (25.0)	5907 (18.8)	2154 (15.9)
No	43 632 (80.9)	6701 (75.0)	25 498 (81.2)	11 433 (84.1)
Alcohol consumption [*n*(%)]				
Yes	22 483 (41.7)	3711 (41.5)	13 274 (42.3)	5498 (40.5)
No	31 441 (58.3)	5221 (58.5)	18 131 (57.7)	8089 (59.5)
Exercise habit [*n*(%)]				
Yes	37 855 (70.2)	6835 (76.5)	21 204 (67.5)	9816 (72.2)
No	16 069 (29.8)	2097 (23.5)	10 201 (32.5)	3771 (27.8)
Sleep quality [*n*(%)]				
Very poor	2583 (4.8)	352 (3.9)	1394 (4.4)	837 (6.2)
Poor	6396 (11.9)	821 (9.2)	3638 (11.6)	1937 (14.3)
Fair	29 768 (55.2)	4796 (53.7)	17 369 (55.3)	7603 (56.0)
Good	12 272 (22.8)	2311 (25.9)	7337 (23.4)	2624 (19.3)
Very good	2905 (5.4)	652 (7.3)	1667 (5.3)	586 (4.3)
Underlying disease [*n*(%)]				
Yes	19 096 (35.4)	3685 (41.3)	9667 (30.8)	5744 (42.3)
No	34 828 (64.6)	5238 (58.7)	21 738 (69.2)	7843 (57.7)
Distance to the nearest medical facility [*n*(%)]				
Less than 1 km	17 591 (32.6)	3039 (34.0)	10 046 (32.0)	4506 (33.2)
1–3 km	23 286 (43.2)	3755 (42.0)	13 746 (43.8)	5785 (42.6)
3 km and further	13 047 (24.2)	2138 (23.9)	7613(24.2)	3296 (24.3)
Service utilization affected by COVID-19 [*n*(%)]				
Yes	19 462 (36.1)	2899 (32.5)	9551 (30.4)	7012 (51.6)
No	34 462(63.9)	6033(67.5)	21 854(69.6)	6575(48.4)
Depressive symptoms [mean (SD)]	6.01 (6.43)	4.76 (6.24)	5.67 (6.17)	7.63 (6.84)

Percentages may not total 100 because of rounding

Table 2 presents COVID-19 infection and symptoms in all infected participants. Infection was defined by a positive result of nucleic acid test or antigen test. A total of 44 992 participants (83.44%) reported contracting COVID-19 at least once, with 0.6% experiencing three or more infections. Among those infected, 72.3% experienced related symptoms for up to 10 days, while 16.8%, 7.9% and 3.0% reported symptoms for 11–20 days, 21–30 days and over 30 days, respectively. The reported severity of the COVID-19 symptoms varied from asymptomatic (4.2%) to mild (57.2%), moderate (37.1%) and severe (1.5%). Additionally, 33.6% of those infected expressed an intent to seek medical attention for their symptoms.

**Table 2:** COVID-19 infection and symptoms of all infected, infected but not utilizing health services, infected and utilizing health services participants

Health service utilization			
Characteristic	Infected		
	Total	Not utilizing health services	Utilizing health services
*n*	44 992	31 405	13 587
Frequency of infection [*n*(%)]			
Once	44 452 (98.8)	31 128 (99.1)	13 324 (98.1)
Twice	273 (0.6)	124 (0.4)	149 (1.1)
Three times or more	267 (0.6)	153 (0.5)	114 (0.8)
Number of days duration of symptoms [*n*(%)]			
10 days or less	32 522 (72.3)	24 027 (76.5)	8495 (62.5)
11–20 days	7575 (16.8)	4892 (15.6)	2683 (19.7)
21–30 days	3572 (7.9)	1929 (6.1)	1643 (12.1)
More than 30 days	1323 (3.0)	557 (1.8)	766 (5.6)
Severity of symptoms [*n*(%)]			
Asymptomatic	1880 (4.2)	1283 (4.1)	597 (4.4)
Mild	25 727 (57.2)	19 019 (60.6)	6708 (49.4)
Moderate	16 694 (37.1)	10 981 (35.0)	5713 (42.0)
Severe	691 (1.5)	122 (0.4)	569 (4.2)
Intention to seek medical services [*n*(%)]			
Yes	15 122 (33.6)	5339 (17.0)	9783 (72.0)
No	29 870 (66.4)	26 066 (83.0)	3804 (28.0)

Percentages may not total 100 because of rounding

From among the surveyed population, 13 587 individuals utilized health services due to COVID-19. Of these cases, 0.8% experienced three or more infections, 5.6% manifested symptomatology for more than 30 days, 4.2% presented severe symptoms and 72.0% intended to seek medical attention for COVID-19 symptoms.

### Health service utilization due to COVID-19

**Fig. 1:**
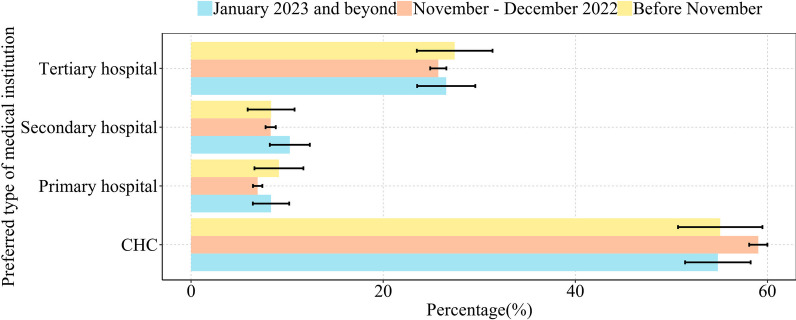
Distribution of the type of medical institution preferred by infected participants over time

Table 3 presents health service utilization due to COVID-19. In the healthcare service utilization due to COVID-19, the majority (85.6%) of individuals chose in-person healthcare, with the remaining 14.4% choosing internet-based healthcare. Among participants who received in-person health services, 82.2% choose to visit the nearest healthcare institution. A larger proportion of these participants (58.6%) reported visiting primary healthcare institutions compared with tertiary hospitals (25.9%). For the infected population, we partitioned them into distinct time periods: pre-outbreak (before November 2022), outbreak peak (November–December 2022) and post-outbreak (January 2023 and beyond). Figure 1 shows the distribution of chose type of medical institution utilized during each time frame. It was noted that 59.04% of participants chose primary healthcare institution as their initial medical institution during November–December 2022, surpassing the percentages observed in the pre-outbreak period (55.08%) and post-outbreak period (54.83%).

**Table 3:** Characteristics of health service utilization due to COVID-19

Characteristic	Internet-based healthcare		In-person healthcare
*n*(%)	1950 (14.4)		11 637 (85.6)
Severity of symptoms [*n*(%)]			
Asymptomatic	198 (10.2)		399 (3.4)
Mild	911 (46.7)		5797 (49.8)
Moderate	784 (40.2)		4929 (42.4)
Severe	57 (2.9)		512 (4.4)
Days to first healthcare seeking with symptoms [*n*(%)]			
Day 1	444 (22.8)		2111 (18.1)
Day 2	460 (23.6)		2510 (21.6)
Day 3	286 (14.7)		1657 (14.2)
Day 4–5	127 (6.5)		866 (7.4)
More than 5 days	168 (8.6)		2780 (23.9)
Unclear	465 (23.8)		1713 (14.7)
Preferred type of medical institution [*n*(%)]			
Primary healthcare institution	N/A		6817 (58.6)
First-level hospital	N/A		829 (7.1)
Secondary hospital	N/A		981 (8.4)
Tertiary hospital	N/A		3010 (25.9)
Choosing the nearest medical institution [n(%)]			
No	N/A		2076 (17.8)
Yes	N/A		9561 (82.2)
Waiting time for medical treatment [*n*(%)]			
Within 15 min	849 (43.5)		3251 (27.9)
15–30 min	452 (23.2)		3223 (27.7)
30–60 min	257 (13.2)		2209 (19.0)
1–2 h	177 (9.1)		1535 (13.2)
2–4 h	78 (4.0)		763 (6.6)
4–6 h	40 (2.1)		317 (2.7)
More than 6 h	97 (5.0)		339 (2.9)
Require for hospitalization [*n*(%)]			
No	N/A		11 446 (98.4)
Yes	N/A		191 (1.6)
Clinical classification [*n*(%)]			
Light	939 (48.15)		4623 (39.73)
Regular	577 (29.59)		3421 (29.40)
Severe	65 (3.33)		266 (2.29)
Critical	9 (0.46)		53 (0.46)
No clinical classification	360 (18.46)		3274 (28.13)
Improvement in symptoms after treatment [*n*(%)]			
Cured	750 (38.5)		2931 (25.2)
Significantly improved	569 (29.2)		4152 (35.7)
Slightly improved	464 (23.8)		3485 (29.9)
Not improved	155 (7.9)		1038 (8.9)
Worsened	12 (0.6)		31 (0.3)
Satisfaction with treatment services [*n*(%)]			
Not satisfied	142 (7.3)		187 (1.6)
Not very satisfied	84 (4.3)		372 (3.2)
Basically satisfied	599 (30.7)		2929 (25.2)
Satisfied	520 (26.7)		3325 (28.6)
Very satisfied	605 (31.0)		4824 (41.5)

*N/A* not applicable. Percentages may not total 100 because of rounding

The proportion of patients necessitating hospitalization due to severe COVID-19 symptoms was relatively low, representing only 1.6%. Interestingly, more than a quarter (27.9%) of participants seeking treatment at healthcare institutions reported waiting for less than 15 min, while a small percentage (5.6%) had to wait for 4 h or longer. Conversely, among those who sought internet-based healthcare, a significant proportion (43.5%) experienced a waiting time of under 15 min, but a non-negligible subset (7.1%) still had to wait for more than 4 h. Participants who experienced wait times exceeding 4 h for both internet-based and in-person healthcare were predominantly female, aged from 31 to 45 years and exhibiting moderate symptoms of COVID-19 (see detailed information in Additional file 1: Table S4). Among individuals seeking in-person healthcare due to COVID-19, 1.6% required hospitalization; 2.29% exhibited a clinical classification as severe, while 0.46% were categorized as critical cases. In contrast, for those opting for internet-based healthcare, 3.33% demonstrated a severe clinical classification, with 0.46% falling into the critical category. We also examined the improvement in symptoms after treatment, revealing that online medical consultation boasted a self-reported cure rate of 38.5% compared with the in-person medical treatment self-reported cure rate of 25.2%. A total of 10.2% of the population receiving online medical consultations had asymptomatic symptoms, while 2.9% had severe symptoms. Furthermore, 4.4% of patients who visited medical institutions for treatment showed severe symptoms, compared with 3.4% of patients who had asymptomatic symptoms. These findings indicated a potential correlation between symptom severity and the form of health consultation, and people choosing online medical consultation generally presented milder symptoms. In terms of satisfaction with treatment, 41.5% of those who seeking treatment at a healthcare institution reported being very satisfied, while 1.6% were dissatisfied. Conversely, among those who chose internet-based healthcare, 31.0% reported being very satisfied, but 7.3% reported dissatisfaction.

### Association of socio-demographic indicators, infection status and depression with health service utilization due to COVID-19

**Fig. 2:**
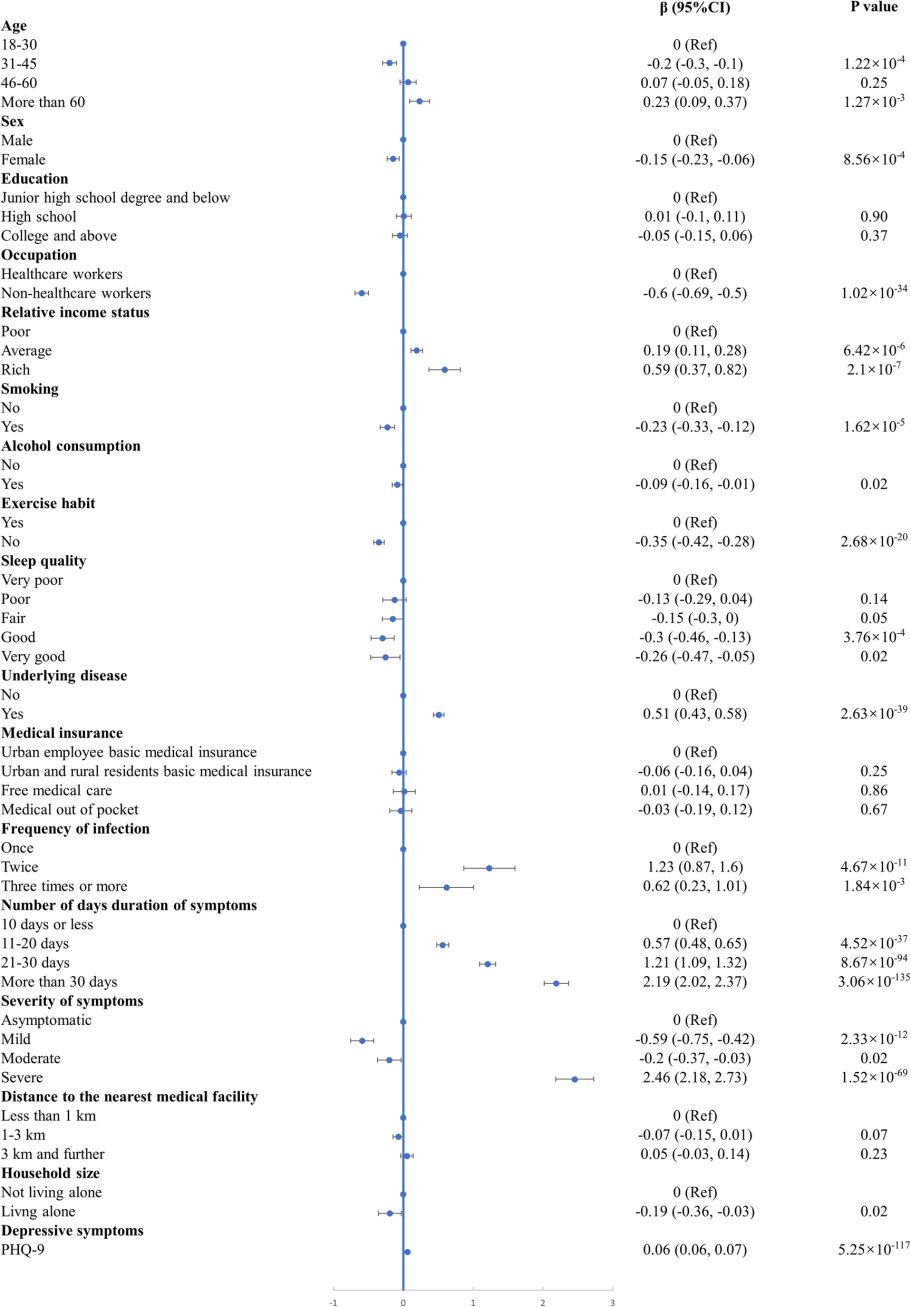
Results of Tobit regression for factors influencing health service utilization due to COVID-19

The Tobit regression analysis results are summarized in Fig. 2. After controlling for confounders, several factors were found to have a statistically significant association with health service utilization due to COVID-19. Compared with participants aged 18–30 years, those aged 31–45 years reported lower frequencies (*β* = −0.20,* P* < 0.001) of health service utilization due to COVID-19, while those aged more than 60 years sought health services more frequently (*β* = 0.23, *P* < 0.01). Non-healthcare workers sought treatment for COVID-19 less often compared with healthcare workers (*β* = −0.60, *P* < 0.001). Participants utilized health services more frequently if they had fair (*β* = 0.19, *P* < 0.001) or rich (*β* = 0.59, *P* < 0.001) self-rated income level. Participants who reported smoking (*β* = −0.23, *P* < 0.001) and drinking alcohol (*β* = −0.09, *P* < 0.05) were both less likely to utilize health service. Additionally, individuals without exercise habits (*β* = −0.35, *P* < 0.001) sought medical care less often than those with exercise habits. Female participants (*β* = −0.15, *P* < 0.001) and those who reported good (*β* = −0.30, *P* < 0.001) or very good (*β* = −0.26, *P* < 0.05) sleep quality utilized health services less often. Furthermore, living alone was associated with lower healthcare utilization (*β* = −0.19, *P* < 0.05), while having underlying diseases was associated with higher healthcare utilization (*β* = 0.51, *P* < 0.001).

Participants were found to be more likely to seek health services if they were infected with COVID-19 more than once or experienced symptoms lasting for longer than 10 days. Compared with asymptomatic individuals, those with mild (*β* = −0.59, *P* < 0.001) and moderate (*β* = −0.20, *P* < 0.05) symptoms sought medical attention significantly less often, while those with severe symptoms sought medical attention more often (*β* = 2.46, *P* < 0.001). Participants reporting higher levels of depressive symptoms were more likely to receive healthcare services due to COVID-19 (*β* = 0.06, *P* < 0.001).

## Discussion

COVID-19 has had a significant impact on everyone’s life, and can particularly affect the health service utilization in the general public. It was likely that some people were more affected than others. This study explored the health service utilization patterns among patients with COVID-19 with different conditions and the related factors after relaxation of the dynamic zero-COVID policy. A better understanding of factors associated with health service utilization due to COVID-19 among residents will help to improve the health care delivery system as well as the dynamic emergency response mechanism.

In the context of a substantial rise in COVID-19 cases, online medical consultations commonly resulted in prescriptions for antipyretic and antiviral medications. Notably, medications could be prescribed during the first visit. We found that people continue to prefer seeking health services from healthcare institutions, with less than 15% of infected individuals opting for online medical consultations. This is consistent with a previous survey conducted in Wuhan, in which people preferred face-to-face health services over online consultations [21]. The vast majority of participants who received online consultations experienced asymptomatic and mild symptoms. Online medical consultations can provide access to subspecialists who are not immediately available in person [22]. With the promotion of digital health in China during the last few years, many medical institutions have started online health services with increasing acceptability by the population, while it still requires time to develop. Satisfaction with treatment of online consultations was relatively low, possibly owing to lengthy waiting times, uncertainty regarding the timing of physician responses, and inadequate resolution of urgent health issues.

We observed that a considerable number of patients chose primary healthcare institutions as their initial point of medical care. This upsurge in the number of infections has been driven largely by the Omicron subvariant [23], which predominantly induces mild symptoms that can be adequately addressed by primary healthcare services, such as prescription of symptomatic medicines. The proportion of infected individuals seeking medical treatment at primary healthcare institutions was the highest during November–December 2022. In December 2022, the Chinese government prioritized the strengthening of primary healthcare systems by facilitating equitable distribution of medical resources (particularly antiviral drugs) to primary healthcare institutions [7]. Such measures are aimed at promoting trust and utilization in primary healthcare institutions, so as to promote the implementation of tiered diagnosis and treatment. Our study affirms that this goal is being advanced to some extent.

Several socio-demographic characteristics and the symptom profiles of COVID-19 were significantly related to health service utilization patterns in the current study. Specifically, individuals aged 61 years or older and those with underlying medical conditions reported higher rates of healthcare utilization in response to COVID-19, underlining their heightened need for medical attention stemming from poor health status. Participants with healthier lifestyle habits (such as no smoking, no alcohol consumption or regular exercise) and better sleep quality exhibited a stronger inclination towards prioritizing their health and demonstrate higher healthcare utilization rates due to COVID-19. These findings are consistent with previous studies [24]. However, some studies identified exercise and non-smoking as protective factors against COVID-19 infection, which might lead to decreased healthcare utilization [25]. Furthermore, non-living alone individuals tend to seek medical attention more frequently due to COVID-19 than those living alone, perhaps due to concerns about transmitting the virus to other family members.

We found that participants with fair and rich self-rated incomes were more likely to utilize health services due to COVID-19 compared with participants with poor self-rated incomes, and that rich individuals were more likely than fair individuals. A study of health service utilization in northeastern Ethiopia found that participants above the poverty line were 4.026 times more likely to use health services than those below the poverty line [26]. Other studies had similar findings [27, 28], indicating that individuals with better economic statuses are more capable of affording the direct and indirect expenses associated with health service utilization and benefit more from health service utilization.

Individuals who have experienced multiple COVID-19 infections or prolonged symptom duration reported higher healthcare utilization rates. A notable observation is that individuals with mild or moderate symptoms exhibit less frequent healthcare service utilization due to COVID-19 than asymptomatic individuals, while those with severe symptoms tend to seek medical attention more often. This is possibly due to asymptomatic individuals having better physical conditions to complete healthcare behaviours and being more willing and physically capable of stockpiling medication than those with milder symptoms. Conversely, those with severe symptoms must visit medical institutions for professional treatment.

We also identified a positive correlation between greater levels of depression symptoms and increased healthcare utilization rates in response to COVID-19. This trend may partly rely on the hypochondriasis effect, whereby individuals suffering from depression disproportionately perceive their health status as extremely poor, leading them to seek medical attention at even minor signs of discomfort and find psychological reassurance through receiving healthcare services. These findings align with results from previous research, in which depression was associated with markedly increased healthcare utilization [29, 30]. We found a positive association between depression symptoms with healthcare utilization due to COVID-19 for the first time.

There is uncertainty among some studies regarding the ability of China's healthcare system to handle a large-scale COVID-19 outbreak [3]. According to the collected questionnaire responses in this study, the majority of surveyed residents in Beijing reported satisfaction with the health services received, and most medical institutions have taken prompt action to allocate their medical staff accordingly to effectively manage the increase of patient demand. As the COVID-19 epidemic enters a frequent, less deadly waves pattern [31], it is important for healthcare systems to ensure access to safe and reliable care, particularly for vulnerable populations such as the elderly, those with underlying diseases and individuals with depressive symptoms. To prepare for unexpected emergencies, medical institutions should establish emergency preparedness plans that enable flexible allocation of manpower, equipment and medication. According to the actual medical needs of residents, governments should gradually improve the medical environment and service capacity of primary healthcare institutions, reduce their gap with higher-level hospitals and attract patients seeking medical treatment in primary healthcare institution first. The current allocation of antiviral drugs to primary healthcare institutions on a priority basis works well in Beijing. Additionally, online medical services should be strengthened. These play a very important role in the rational allocation of medical resources and optimize the efficiency of resource utilization.

Our study had several limitations. Firstly, the data from the online survey was cross-sectional, which may inherently restrict our ability to infer any causal relationships and only allows us to describe medical care-seeking behaviours of the population at a particular point of time. It is important to note that our study was conducted during a period when COVID-19 infections were on the rise, following the relaxation of prevention and control measures in China. Additionally, the questionnaire had some questions that involve subjective feelings, which may have recall bias and make the results less accurate. Secondly, the distribution of our questionnaire by doctors affiliated with primary healthcare institutions may have influenced contracted residents to exhibit a higher propensity towards seeking health services, particularly primary healthcare services. Consequently, this could have resulted in an increased proportion of individuals seeking medical treatment in primary healthcare institutions compared to the general population, potentially introducing a sample selection bias. Additionally, we recognize that the observed gender and age imbalances in our study can be attributed to factors such as the voluntary nature of participation and the preference for cooperative behaviour among female participants. The use of the Wenjuanxing platform may have contributed to the imbalance, as the elderly population might be less familiar with smartphones and electronic survey platforms. Lastly, the healthcare response in Beijing was different from other regions, limiting the extrapolation of our findings to other regions.

## Conclusions

COVID-19 has disrupted regular health service utilization, requiring new approaches to meeting the needs of residents, especially following the relaxation of the dynamic zero-COVID policy. Age of 60 years or older, being male, higher income, healthy lifestyle habits, underlying diseases, not living alone, severe depressive symptoms, longer symptom duration, more infections and asymptomatic or severe symptoms were significant predictors for health service utilization due to COVID-19. Medical providers can utilize these findings to comprehend patients’ concerns during such times and create effective policies that cater to various needs.

### Supplementary Information

**Additional file 1: Table S1.** Classification of demographics and health-related indicators. **Table S2.** Classification of COVID-19 infection and symptoms indicators. **Table S3.** Classification of health service utilization indicators. **Table S4.** Characteristics of participants who waited more than 4 h for health service utilization due to COVID-19.

## Data Availability

The datasets used during the study are available from the corresponding author on request.

## Abbreviations

COVID-19: Coronavirus disease 2019
PHQ-9: Patient Health Questionnaire-9
