# Supplementary material for: Analysis of health service utilization and influencing factors due to COVID-19 in Beijing: a large cross-sectional survey
Source: Health Res Policy Syst. 2024 Mar 4;22:31. doi: 10.1186/s12961-024-01118-6 (PMC10910832; doi:10.1186/s12961-024-01118-6)
Supplement: Supplementary file 1 — Additional file 1: Table S1. Classification of demographics and health-related indicators. Table S2. Classification of COVID-19 infection and symptoms indicators. Table S3. Classification of health service utilization indicators. Table S4. Characteristics of participants who waited more than 4 h for health service utilization due to COVID-19. [file 12961_2024_1118_MOESM1_ESM.docx]

**Additional file 1**

**Analysis of health service utilization and influencing factors due to COVID-19 in Beijing: a large cross-sectional survey**

Table S1 Classification of demographics and health-related indicators.

Table S2 Classification of COVID-19 infection and symptoms indicators

Table S3 Classification of health service utilization indicators

Table S4 Characteristics of participants who waited more than 4 hours for health service utilization due to COVID-19

**Table S1 Classification of demographics and health-related indicators**

| **Indicator** | **Classification** |
| --- | --- |
| Age | 18-30 |
|  | 31-45 |
|  | 46-60 |
|  | older than 60 |
| Gender | female |
|  | male |
| Relative income status | poor |
|  | average |
|  | wealthy |
| Marital status | married |
|  | not married [including unmarried, divorced, and widowed] |
| Education level | junior high school degree and below |
|  | high school degree |
|  | college and above |
| Occupation type | healthcare workers |
|  | non-healthcare workers |
| Medical insurance coverage | urban employee basic medical insurance |
|  | urban and rural residents basic medical insurance |
|  | free medical care |
|  | medical out of pocket |
| Household size | live alone |
|  | not live alone |
| Underlying diseases | yes |
|  | no |
| Sleep quality | very poor |
|  | poor |
|  | fair |
|  | good |
|  | very good |
| Smoking | yes |
|  | no |
| Alcohol consumption | yes |
|  | no |
| Exercise habits | yes |
|  | no |
| The distance to the nearest medical facility from their home | less than 1 km |
|  | 1-3 km |
|  | 3 km and further |
| Influence of COVID-19 on healthcare seeking | yes |
|  | no |

**Table S2** **Classification of COVID-19 infection and symptoms indicators**

| **Indicator** | **Classification** |
| --- | --- |
| Frequency of infection | none |
|  | once |
|  | twice |
|  | three times or more |
| Number of days duration of symptoms | 10 days or less |
|  | 11 to 20 days |
|  | 21 to 30 days |
|  | more than 30 days |
| Severity of symptoms | asymptomatic |
|  | mild |
|  | moderate |
|  | severe |
| Intention of infected people to seek health services for COVID-19 symptoms | yes |
|  | no |

**Table S3 Classification of health service utilization indicators**

| **Indicator** | **Classification** |
| --- | --- |
| Days to first healthcare seeking with symptoms | day 1 |
|  | day 2 |
|  | day 3 |
|  | day 4-5 |
|  | more than 5 days |
|  | unclear |
| Form of health consultation | internet-based healthcare |
|  | in-person healthcare |
| Preferred type of medical institution | CHC |
|  | primary hospital |
|  | secondary hospital |
|  | tertiary hospital |
| Waiting time for medical treatment | within 15 minutes |
|  | 15-30 minutes |
|  | 30-60 minutes |
|  | 1-2 hours |
|  | 2-4 hours |
|  | 4-6 hours |
|  | more than 6 hours |
| Require for hospitalization due to COVID-19 | yes |
|  | no |
| Improvement in symptoms after treatment | cured |
|  | significantly improved |
|  | slightly improved |
|  | not improved |
|  | worsened |
| Satisfaction with treatment services | not satisfied |
|  | not very satisfied |
|  | basically satisfied |
|  | satisfied |
|  | very satisfied |

**Table S4 Characteristics of participants who waited more than 4 hours for health service utilization due to COVID-19**

| Characteristic | Total | Internet-based Healthcare | In-person Healthcare |
| --- | --- | --- | --- |
| n | 793 | 137 | 656 |
| Age(n(%)) |  |  |  |
| 18-30 | 125 (15.8) | 26 (19.0) | 99 (15.1) |
| 31-45 | 328 (41.4) | 63 (46.0) | 265 (40.4) |
| 46-60 | 195 (24.6) | 31 (22.6) | 164 (25.0) |
| Older than 60 | 145 (18.3) | 17 (12.4) | 128 (19.5) |
| Gender (n(%)) |  |  |  |
| Female | 503 (63.4) | 83 (60.6) | 420 (64.0) |
| Male | 290 (36.6) | 54 (39.4) | 236 (36.0) |
| Marital status (n(%)) |  |  |  |
| Married | 687 (86.6) | 114 (83.2) | 573 (87.3) |
| Not married | 106 (13.4) | 23 (16.8) | 83 (12.7) |
| Medical insurance (n(%)) |  |  |  |
| Urban employee basic medical insurance | 627 (79.1) | 97 (70.8) | 530 (80.8) |
| Urban and rural residents basic medical insurance | 109 (13.7) | 23 (16.8) | 86 (13.1) |
| Free medical care | 32 (4.0) | 11 (8.0) | 21 (3.2) |
| Medical out of pocket | 25 (3.2) | 6 (4.4) | 19 (2.9) |
| Education (n(%)) |  |  |  |
| Junior high school degree and below | 161 (20.3) | 27 (19.7) | 134 (20.4) |
| High school degree | 170 (21.4) | 25 (18.2) | 145 (22.1) |
| College and above | 462 (58.3) | 85 (62.0) | 377 (57.5) |
| Relative income status (n(%)) |  |  |  |
| Poor | 247 (31.1) | 45 (32.8) | 202 (30.8) |
| Average | 528 (66.6) | 89 (65.0) | 439 (66.9) |
| Wealthy | 18 (2.3) | 3 (2.2) | 15 (2.3) |
| Frequency of infection (n(%)) |  |  |  |
| Once | 773 (97.5) | 130 (94.9) | 643 (98.0) |
| Twice | 13 (1.6) | 5 (3.6) | 8 (1.2) |
| Three times or more | 7 (0.9) | 2 (1.5) | 5 (0.8) |
| Number of days duration of symptoms (n(%)) |  |  |  |
| 10 days or less | 445 (56.1) | 93 (67.9) | 352 (53.7) |
| 11-20 days | 147 (18.5) | 23 (16.8) | 124 (18.9) |
| 21-30 days | 127 (16.0) | 16 (11.7) | 111 (16.9) |
| More than 30 days | 74 (9.3) | 5 (3.6) | 69 (10.5) |
| Severity of symptoms (n(%)) |  |  |  |
| Asymptomatic | 38 (4.8) | 11 (8.0) | 27 (4.1) |
| Mild | 301 (38.0) | 58 (42.3) | 243 (37.0) |
| Moderate | 345 (43.5) | 60 (43.8) | 285 (43.4) |
| Severe | 109 (13.7) | 8 (5.8) | 101 (15.4) |

Percentages may not total 100 because of rounding.
